# 1115. Evaluation of Gepotidacin (GSK2140944) Pharmacokinetics and Food Effect in Japanese Subjects

**DOI:** 10.1093/ofid/ofab466.1309

**Published:** 2021-12-04

**Authors:** Mohammad Hossain, Courtney Tiffany, Aline Barth, Aparna Raychaudhuri, Etienne F Dumont

**Affiliations:** 1 GlaxoSmithKline plc., Collegeville, PA, USA, Collegeville, Pennsylvania; 2 GSK

## Abstract

**Background:**

Gepotidacin, a novel, first-in-class triazaacenaphthylene antibiotic, inhibits bacterial replication and has *in vitro* and *in vivo* activity against key pathogens, including drug-resistant strains, associated with a range of infections. Gepotidacin is currently in Phase 3 clinical studies for the treatment of uncomplicated urinary tract infections and gonorrhea. This study (NCT02853435) was designed to assess gepotidacin pharmacokinetics (PK) in Japanese subjects (fasted and fed).

**Methods:**

A tablet formulation of 750 mg gepotidacin free base was used in the study, which was conducted in two parts: Part 1, gepotidacin PK was assessed following 1500 and 3000 mg single oral doses in the fasted state; and Part 2, gepotidacin PK was assessed following 1500, 2250, and 3000 mg single oral doses in the fed state. Serial blood and urine samples were collected in both study parts.

**Results:**

Part 1: The area under the plasma drug concentration-time curve from time 0 to infinity (AUC_[0–∞]_) and maximum observed concentration (C_max_) were slightly higher in Japanese subjects than in Caucasian subjects at the same dose levels and with the same formulation. Following gepotidacin dosing in the fasted state, the 1500 mg dose was tolerated, while the 3000 mg dose was poorly tolerated with mild or moderate gastro-intestinal adverse effects (GI AEs) reported by most subjects shortly after being dosed. Part 2: PK was linear with doses in the range of 1500–3000 mg. Administration of gepotidacin 3000 mg tablets in the fed state slightly reduced C_max_ and slightly increased AUC at the 3000 mg dose level. The 1500 and 2250 mg doses were tolerated while the 3000 mg dose was better tolerated compared to the fasted state with fewer and short-lived GI AEs, mostly mild in intensity. After oral administration of 1500–3000 mg, high urine drug concentrations were achieved, remaining above the minimum inhibitory concentration of 4 μg/mL for up to 24 hours.

**Conclusion:**

The PK of gepotidacin following administration of a single oral dose to Japanese subjects was linear from 1500–3000 mg and food decreased C_max_ without impact on exposure (AUC). Administration of gepotidacin with food resulted in an improved GI tolerability profile at the higher dose tested in Japanese subjects.

**Disclosures:**

**Mohammad Hossain, PhD**, **GlaxoSmithKline plc.** (Employee, Shareholder, Former employee of and past/current shareholder in GlaxoSmithKline plc.) **Courtney Tiffany, BSc**, **GlaxoSmithKline plc.** (Employee, Shareholder, Former employee of and past/current shareholder in GlaxoSmithKline plc.) **Aline Barth, MSC;PHD**, **GlaxoSmithKline plc.** (Employee, Shareholder, Employee of and shareholder in GlaxoSmithKline plc.) **Aparna Raychaudhuri, Ph.D.**, **GlaxoSmithKline plc.** (Employee, Shareholder, Former employee of and past/current shareholder in GlaxoSmithKline plc.) **Etienne F. Dumont, MD**, **GlaxoSmithKline plc.** (Employee, Shareholder, Former employee of and shareholder in GlaxoSmithKline plc.)

